# Leveraging Dual Usability Methods to Evaluate Clinical Decision Support Among Patients With Traumatic Brain Injury: Mixed Methods Study

**DOI:** 10.2196/60268

**Published:** 2025-07-30

**Authors:** Rubina F Rizvi, Sameen Faisal, Mark Sussman, Patricia Mendlick, Sam Brown, Elizabeth Lindemann, Sean Switzer, Genevieve B Melton, Christopher J Tignanelli

**Affiliations:** 1 Department of Surgery, University of Minnesota, 420 Delaware Street South East, Mayo Mail Code 195, Minneapolis, MN, 55455, United States, 1 612-626-1999; 2 Center For Learning Health System Sciences, University of Minnesota, Minneapolis, MN, United States; 3 Institute for Health Informatics, University of Minnesota, Minneapolis, MN, United States; 4 College of Biological Sciences, University of Minnesota, Minneapolis, MN, United States; 5 Stanson Premier Inc, Charlotte, NC, United States; 6 Fairview Health Services, Minneapolis, MN, United States

**Keywords:** traumatic brain injury, venous thromboembolism, clinical decision support system (CDSS), heuristic evaluations, end user–based usability testing, decision support system

## Abstract

**Background:**

Patients with traumatic brain injury are at an increased risk of developing venous thromboembolism. Clinical decision support systems (CDSSs) may improve the use of venous thromboembolism prophylaxis protocols, yet suffer from poor compliance among end users due to a lack of user-centered design.

**Objective:**

The objective of this work was to improve the content, design, and workflow integration of a traumatic brain injury–CDSS based on feedback from experts and end users.

**Methods:**

The CDSS was evaluated leveraging a dual usability approach. A set of usability experts (n=3) and trauma providers (n=5) performed heuristic evaluations and usability testing by end users. Data was collected through a triangulation of methods and analyzed using qualitative (thematic) and quantitative (descriptive) analyses.

**Results:**

Among the 145 total issues identified across both methods, 66 issues were found to be unique. Of the 66, a total of 17 issues were found by heuristic evaluations, 43 by usability testing by end users, and 6 were found across both methods. Thematic analysis was conducted on the 66 unique issues, which were further assigned to themes and subsequent subthemes. We identified 13 unique themes. The 3 most prevalent themes of 66 issues were lack of supporting evidence (n=17, 26%), operational barriers arising from the test environment (n=11, 17%), formatting inconsistencies, and lack of following standards (n=8, 12%). The system’s usability scale survey score was 77.5 (SD 16, 95% CI 57.6-97.4), interpreted as an acceptable or good usability range. The mean response score for Single Ease Questions for all tasks was 5.9 (SD 0.53).

**Conclusions:**

Combining expert and end user–driven usability evaluation methods identified a more comprehensive list of issues. This can facilitate the optimization of the traumatic brain injury–CDSS, resulting in improved usability and care management.

## Introduction

Traumatic brain injury is one of the leading causes of mortality and disability worldwide. In the United States alone, over 69,000 traumatic brain injury–related deaths occurred in 2021 [1]. Patients with traumatic brain injury are at an increased risk of developing complications secondary to venous thromboembolism [2]. Fifty-four percent of patients with a traumatic brain injury will develop a venous thromboembolism in the absence of appropriate anticoagulation intervention [3]. Despite the significance of timely venous thromboembolism prophylaxis administration, there has been a reluctance to implement anticoagulation in such patients, thus resulting in delays in timely intervention [4-7].

Optimal use of venous thromboembolism prophylaxis protocols manages the risk of thromboembolism with the competing risk of progression of intracranial bleeding in patients following trauma [7]. However, there is variability in venous thromboembolism prophylaxis protocols across trauma centers [2,7,8]. In an attempt to optimize thromboembolism prophylaxis for the patient, institutional implementation of anticoagulation protocols has been shown to improve clinical outcomes [2,7,8]. To improve prophylactic anticoagulation of patients with traumatic brain injury without adversely affecting outcomes, integrating a clinical decision support system (CDSS) has emerged as an indispensable solution to supplement providers with evidence-based venous thromboembolism prevention guidelines [9]. Existing literature cites improved patient outcomes when evidence-based practices are followed, along with improved compliance with these practices through the use of CDSSs [10-12].

CDSSs, whether stand-alone or integrated with an electronic health records system, are considered a monumental step forward in health care delivery as they serve as reliable and readily available aids to decision makers. CDSSs serve as a platform for clinicians by comparing individual patient data and integrating evidence-based recommendations into their daily workflow [13,14]. When adhered to, these tools serve as an effective means of changing clinician behavior [15-17]. The use of CDSSs varies by purpose and context. However, the resultant benefits of CDSSs are found to consistently positively impact various elements of health care delivery for both patients and clinicians. For providers, it facilitates overall disease management by aiding complex decision-making that is mandated for accurate diagnosis, effective treatment, and efficient monitoring [18]. For patients, the use of CDSSs leads to fewer errors, improved patient safety, improved care quality, and enhanced health outcomes [19].

Despite these benefits, there are some risks and resistance to using CDSS [19]. This includes but is not limited to cost-related challenges, alert fatigue, physicians’ autonomy and trust, liability issues, workflow disruptions, and usability challenges. The negative consequences of a tool to be developed and implemented could be mitigated if iterative evaluations are carried out earlier rather than in the later phase of the system development life cycle [20]. Often, due to the additional resources required (both time and money), iterative evaluations are not prioritized before deployment in clinical practice. However, predeployment usability evaluation could potentially save time and money by preventing the need for critical changes after system implementation [21]. This study highlights the criticality of early testing and engaging end users during the system development life cycle. Identifying and addressing users’ concerns around tool optimization and workflow integration should be considered an integral component when building, implementing, and maintaining a CDSS.

Lack of user-centered design, interoperability, and implementation barriers hinder the widespread adoption of a CDSS [22-24]. There is a need for robust usability studies to address existing barriers and optimize the functionality of the CDSS according to users’ needs. The objective of this study was to evaluate and optimize the usability of a traumatic brain injury CDSS integrated into a US Midwest American College of Surgeons–verified trauma center. Usability issues in a traumatic brain injury-venous thromboembolism-CDSS were identified by leveraging a dual usability evaluation approach. We hypothesized that combining expert and end user–driven methods of usability evaluation would help identify a wide range of unique issues specific to each method, along with common findings shared between the 2 methods.

## Methods

### Overview of Traumatic Brain Injury–Venous Thromboembolism Prevention Guidelines

**Figure 1.**
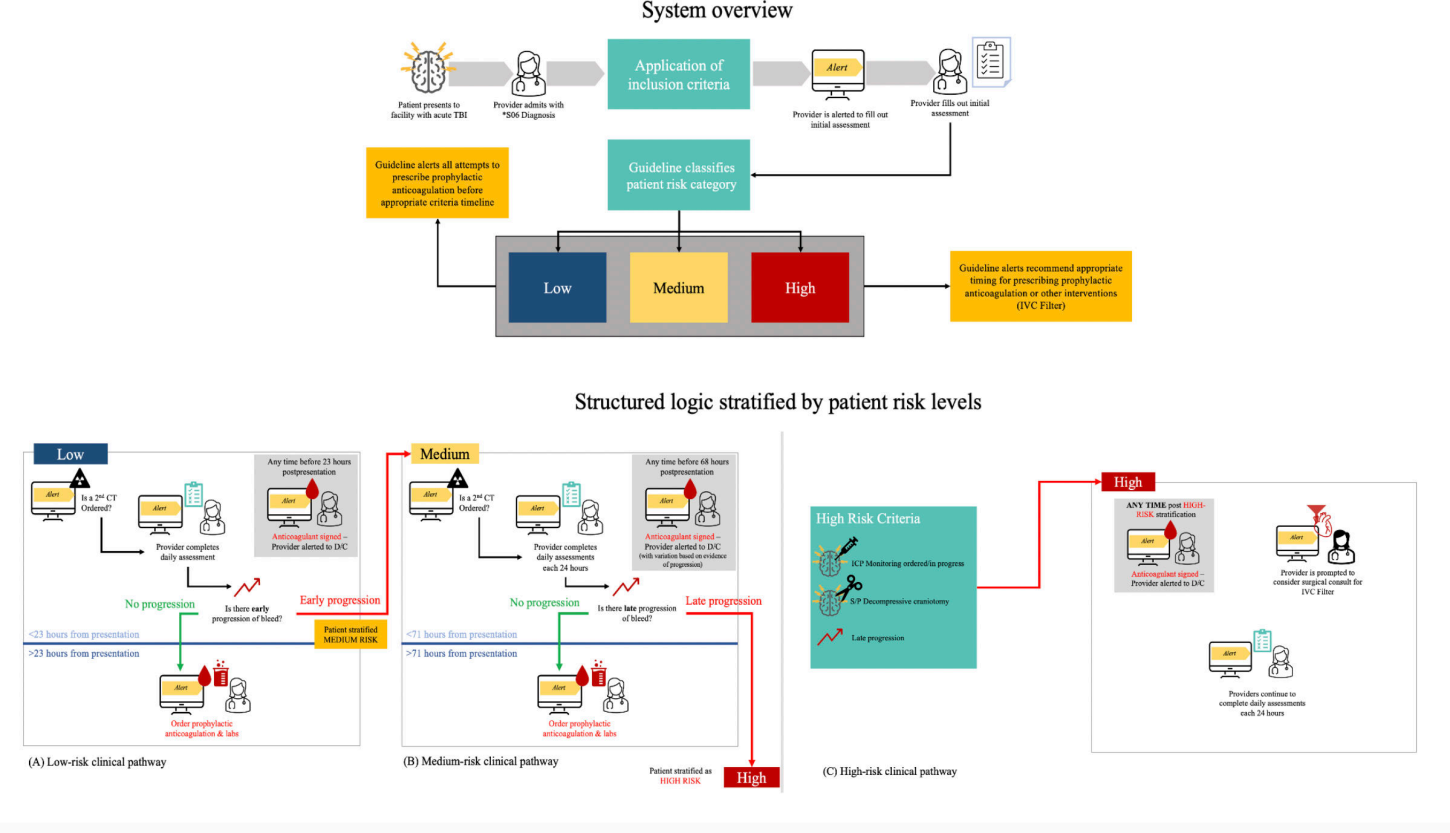
Clinical decision support system logic model for venous thromboembolism prevention among patients with traumatic brain injury. *S06: 2024 *ICD-10-CM* Diagnosis Code S06. CT: computed tomography; D/C: discontinue anticoagulation order; *ICD-10-CM*: *International Statistical Classification of Diseases, Tenth Revision, Clinical Modification*; ICP: intracranial pressure; IVC: inferior vena cava; S/P: status post; TBI: traumatic brain injury.

The traumatic brain injury–CDSS delivers a patient-centered outcomes research guideline for venous thromboembolism prevention in patients with traumatic brain injury [2,25], which was centered around the modified Berne-Norwood criteria [8]. Patients are categorized as being at low, moderate, or high risk for spontaneous progression of hemorrhage, with prophylactic treatment regimens tailored for each group, respectively. The traumatic brain injury–CDSS is a modification of an original, natively developed COVID-19 CDSS logic model, which is currently translated to interoperable standards [16] (Figure 1). We chose this model for two major reasons: (1) we have previous experience implementing the traumatic brain injury venous thromboembolism prevention guideline with success [2], and (2) many of the data elements and artifacts (~80%) overlap with minimal additional mappings and technology build. The final CPG-FHIR (clinical practice guidelines on Fast Healthcare Interoperability Resources) COVID-19 CDSS included 108 data elements. The traumatic brain injury clinical practice guideline added 21 data elements specifically related to traumatic brain injury (eg, intracranial pressure monitoring, neurosurgical consultation, craniotomy, head computed tomography imaging, subdural hematoma, contusion, and Glasgow Coma Scale).

Given the logic model, patients will enter the system by admission under predetermined diagnoses, generally falling under the traumatic brain injury category. Our target population is adult patients admitted with an acute traumatic brain injury, as defined by *ICD-10-CM* (*International Statistical Classification of Diseases, Tenth Revision, Clinical Modification*): S06.1-S06.9 or S06.A. Patients who died within 24 hours of hospital admission and patients documented as “comfort cares” during the first 72 hours of hospitalization will be excluded. Additionally, patients with a pre-existing venous thromboembolism or inferior vena cava filter at the time of admission and patients with a mechanical heart valve or ventricular assist device will be excluded from the final analysis.

The CDSS is provided in the subsequent 72 hours (dependent on risk stratification) and administered in the form of seven alerts (Textbox 1). Textbox 1 provides descriptions of the seven alerts deployed at various points in time, stratified by level of risk.

Textbox 1. **Description of seven alerts in traumatic brain injury–clinical decision support system (CDSS).**

- **Initial assessment:** provides initial risk stratification.
- **Follow-up computed tomography (CT) scan alert:** Guide proper timing of a follow-up CT head scan based on stratification

- **Daily assessments:** Track patient course and modify stratification in the event of critical conditions; that is, progression of bleed, placement of intracranial pressure (ICP) monitor, decompressive craniotomy, etc.
- **Anticoagulation alert:** Guides the health care provider to prescribe the correct dose of anticoagulation medication at the appropriate time, and not too soon.
- **Discontinue anticoagulation order alert:** Any attempt to prescribe anticoagulation before the appropriate clinical decision support system calculated time triggers an alert to discontinue the medication.
- **Laboratory order alert:** Prompts order of appropriate laboratories to monitor side effects of the anticoagulation medications, that is, Anti-Xa, platelets, etc.
- **Laboratory level alerts:** Additional prompts to monitor laboratory levels for appropriateness.

### Study Settings

**Figure 2.**
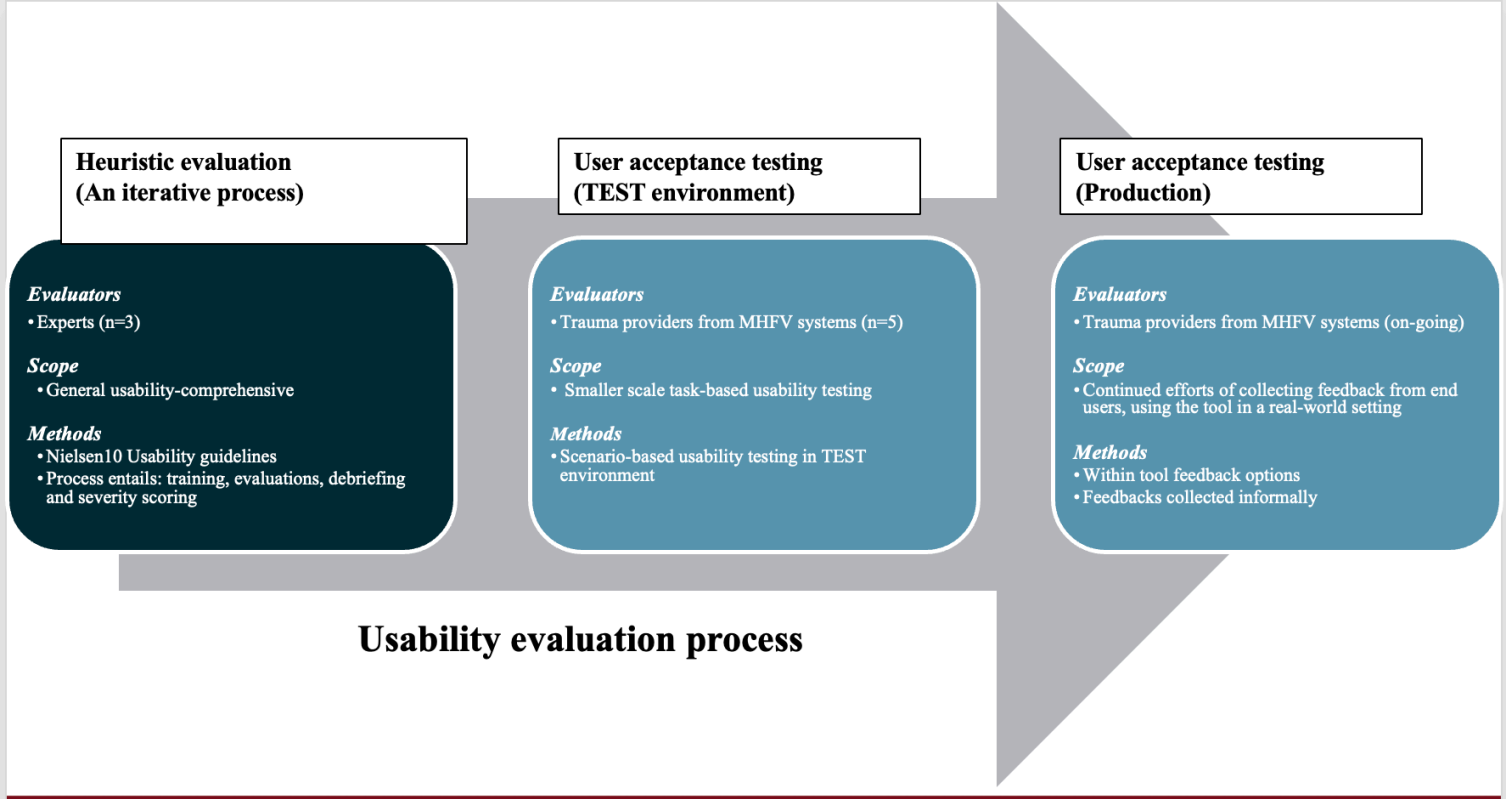
A process diagram of the methodology. MHFV: M (Minnesota) Health Fairview; TEST: test environment.

Our foundational usability evaluation-related research work was conducted by a team of researchers from the University of Minnesota (UMN) and associated health systems, such as the M (Minnesota) Health Fairview and affiliated hospitals, which consisted of 9 state-verified trauma centers, including the flagship UMN as an American College of Surgeons–verified level 2 trauma center. The usability evaluation methodology is reported in Figure 2.

### Data Collection

#### Expert-Based Usability Evaluations

Heuristic evaluations leveraging Nielsen 10 Usability Heuristics for User Interface Design were used iteratively during the whole development cycle, where issues identified were addressed sequentially [26]. The initial round of heuristic evaluations was conducted on screenshots of the user interface that researchers independently evaluated.

Three experts with varied backgrounds, that is, a physician informatics or usability expert (RFR); a hospitalist with content and CDSS-related expertise (SS); and an undergraduate in neuroscience (SF) conducted the evaluation independently. We then had a debriefing session to walk through findings from individual users, removing any duplicates. Once we had a final, consolidated, clean list of unique issues, each evaluator did the severity ranking of each issue. A 0 to 4 rating scale was used to rate the severity of usability problems (0=I do not agree that this is a usability problem at all; 1=cosmetic problem only: need not be fixed unless extra time is available on project; 2=minor usability problem: fixing this should be given low priority; 3=major usability problem: important to fix, so should be given high priority; 4=usability catastrophe: imperative to fix this before product can be released) [27]. While assigning the severity score to usability problems, 3 factors were considered: the frequency with which the problem occurs, the impact of the problem, and the persistence of the problem. The team reconvened in a severity scoring session where a scoring consensus was reached for each problem after mutual deliberations.

#### End Users’ Based Usability Testing

End users’ based usability testing was conducted with actual end users, that is, trauma providers from M Health Fairview and affiliated hospital systems. Purposive sampling was used for recruitment purposes by sharing information about the traumatic brain injury–CDSS usability study through emails, discussions during meetings, word of mouth, etc. Scenario–based usability testing was done with individual end users along with our usability team comprised of up to 5 team members, including a moderator (n=1), facilitators–information technology (IT) experts both from the MHVF and the vendors who helped with building (n=2‐3); and a note taker (n=1). Each session was conducted on Zoom (Zoom Inc.) and lasted up to 90 minutes.

In the scenario-based guided usability testing, we asked users to perform certain tasks on 3 unique test patients corresponding to 3 risk groups: low, medium, and high. Two to four scenarios were associated with each test patient for a total of 9 scenarios, which required each user to perform a few predefined, sequential tasks. Participants were asked to think out loud as they interacted with the traumatic brain injury–CDSS in the Epic testing environment (Epic Inc). Once each scenario and relevant task were completed, participants were asked a few questions to elaborate on their experience and complete a single ease survey questionnaire (REDCap [Research Electronic Data Capture] Consortium; Vanderbilt University). At the end of the session, participants completed a System Usability Scale survey (SUS) [28] and Single Ease Question (SEQ) [29] and were asked to share their overall experience with the tool.

### Building Test Patients and Testing in Epic Test Environment

Our team of experts was comprised of builders or IT specialists (SB, PM, and MS) and clinicians (SS, RFR, and CJT), who helped with building the 3 master test patients stratified by risk level (Textbox 2). Scenarios were generated by a team of clinicians having diverse sets of experiences (ie, CJT, a trauma surgeon, and project principal investigator, SS, a hospitalist, and RFR, a physician informaticist with user acceptance testing experiences) and builders or IT specialists (SB, PM, and MS) after in-depth discussions. The goal was to have scenarios that are commonly encountered and require trauma providers to perform certain high-priority tasks. Copies of the master patients were created to be used by each study participant..

Textbox 2. **Patient risk stratification modified the Berne-Norwood criteria.**

**Test patient # 1: low risk**

- Subdural hematoma 2 mm.
- No moderate or high-risk criteria.

Recommendation: initiate pharmacologic prophylaxis if repeat head computed tomography (CT) was stable at 24 hours.

**Test patient # 2: moderate risk**

- Subdural or epidural hematoma >8 mm.
- Contusion or intraventricular hemorrhage >2 cm.
- Multiple contusions per lobe.
- Subarachnoid hemorrhage with abnormal CT.
- Evidence of progression at 24 hours.
  Recommendation: initiate pharmacologic prophylaxis if the head CT was stable at 72 hours.

**Test patient # 3: high risk**

- Placement of an intracranial pressure monitor, craniotomy, or both.
- Evidence of progression at 72 hours.

Recommendation: consider placement of an inferior vena cava filter.

The purpose of the testing was to receive user feedback effectively and efficiently, ensuring that the testing context matches as much as possible with the user interactions in the real world. Leveraging the Epic test environment to create a simulated environment came with a few constraints described under limitations.

### Data Analysis

#### Overview

The respective data was analyzed both qualitatively and quantitatively as described.

#### Qualitative-Thematic Analysis

We compiled all the issues identified by each method into 1 consolidated list. This included data collected through heuristic evaluations and usability testing by end users. First, a subset of the issue was coded independently by 2 individuals (RFR and SF) at a more granular level, and a codebook was generated after mutual agreement between both coders and applied to the full list of issues. Each analyst coded the rest of the transcripts for various usability problems, using the codebook generated earlier. We cataloged the lowest-level codes into higher-level subthemes and themes to gain a more holistic understanding of traumatic brain injury–CDSS.

#### Quantitative Analysis

The primary focus was on data collected from the SEQ survey and SUS. SEQ is an effective and efficient tool comprised of a single question asked immediately after task completion. SUS is the most widely used and accepted standardized tool to measure subjective usability. We performed descriptive analysis, reporting means and SDs.

### Ethical Considerations

This component of the project is not a clinical trial. This study protocol (STUDY00017107) was submitted to the UMN Institutional Review Board and given the determination of “exempt” as secondary research for which consent is not required. The mixed methods investigation was determined as a “not human research” as a quality improvement activity. Signed and written informed consent was obtained to screen and audio record the session, with the speech-to-text transcription feature turned on. Each recording was saved on a secured and encrypted UMN device. Each participant later received a US $50 gift card for their participation.

## Results

### End Users’ Characteristics

Five trauma providers participated in the individual usability testing session. The detailed characteristics are described in Table 1.

**Table 1.** Demographics of participants.

Characteristics	Trauma providers (n=5), n (%)
Sex	
Male	2 (40)
Female	3 (60)
Role	
MD^a^	4 (80)
APP^b^	1 (20)
Specialty	
Trauma surgery	3 (60)
Neurosurgery	1 (20)
Neurocritical care	1 (20)
Comfort with technology	
Expert	4 (80)
Intermediate	1 (20)

a MD: medical doctor.

b APP: advanced practice provider.

### Usability-Related Findings From the 2 Methods

We identified a set of both common and unique issues using 2 methods: heuristic evaluation with experts (domain experts, ie, physicians, usability experts, or both) and usability testing with end users (the actual users).

### Findings From Heuristics Evaluation

**Figure 3.**
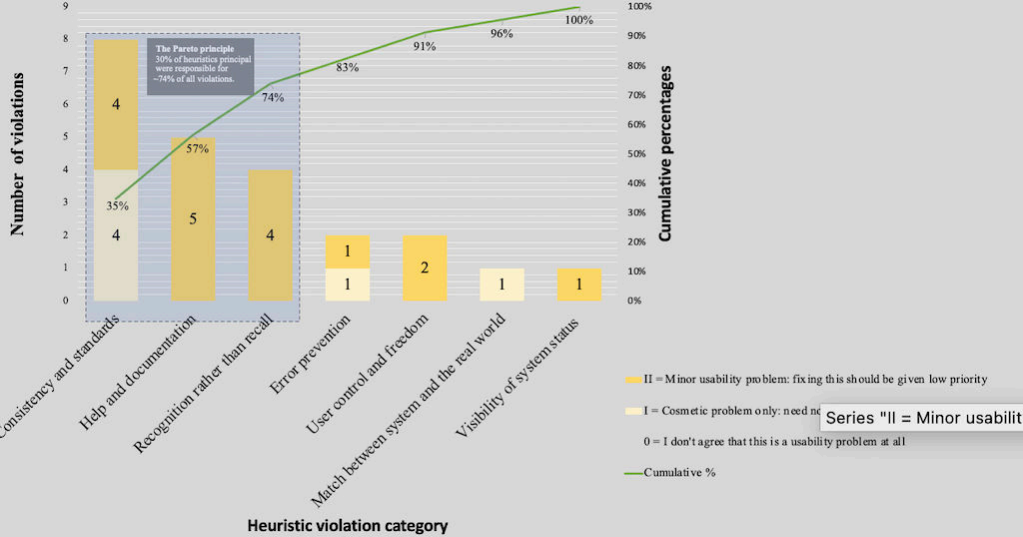
The Pareto chart of heuristics principles violation and the severity ranking.

Thirty-seven heuristic violations (23 unique and 14 duplicates) were identified by 3 experts. The results showed that 30% of possible heuristics were responsible for ~74% of all violations, the Pareto principle (Figure 3). Three heuristics that represented the most violations were (1) consistency and standards (n=8, 35%), (2) help and documentation (n=5, 22%), and (3) recognition rather than recall (n=4, 17%). Of 23 unique, we found cosmetic (n=6) and minor (n=17) issues only.

### Findings From Usability Testing

**Figure 4.**
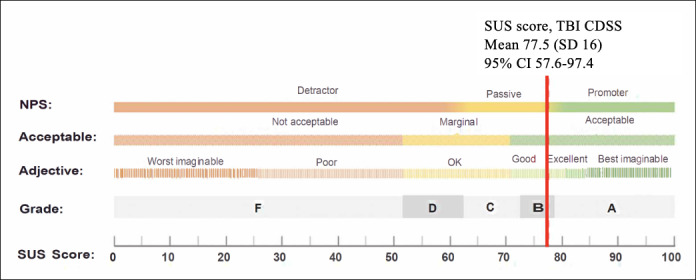
The mean Systems Usability Scale score is interpreted as being within acceptable or good usability range (adapted from Lewis and Sauro [30], with permission from Jim Lewis). CDSS: clinical decision support system; NPS: net promoter score; SUS: Systems Usability Scale survey

We identified 108 issues (49 unique and 59 duplicates). Of 49 issues, we found cosmetic (n=6), minor (n=20), major (n=10), and catastrophic (n=2) issues. The remaining 11 were considered operational and not ranked. The mean responses to SEQ for all tasks were 5.9 (SD 0.53), ranked from 1 (very difficult) to 7 (very easy). The mean SUS score was 77.5 (SD 16) interpreted as an acceptable or good usability range (Figure 4). The 95% CI for the mean SUS score is 77.5 (95% CI 57.6‐97.4).

Between the 2 methods, we identified a total of 145 issues, 66 of which were unique: 17 from heuristic evaluations, 43 from end users, and 6 common issues shared between both methods. Thematic analysis was performed on all unique issues, eliminating those arising from the constraints of thfsuye Epic test environment (66‐11=55).

### Ranking of Usability Issues

We ranked 55 unique usability issues on a severity scale from 0‐4, where 20% (n=11) were marked as cosmetic; 58% (n=32) were identified as minor; 18% (n=10) as major, and 4% (n=2) as catastrophic issues. We identified both cosmetic and minor issues through heuristic evaluations and usability testing with end users. However, severe and catastrophic issues were solely identified during the usability sessions with end users.

### Thematic Analysis

Thematic analysis was conducted on 66 unique issues identified through heuristic evaluations and usability testing. Eleven issues arising from Epic test environment constraints were eliminated. We identified 13 unique themes, including lack of supporting evidence, operational barriers arising from the test environment, formatting inconsistencies and lack of following standards, suboptimal data retrieval and display, lack of user control, lacking language clarity, order set-related suboptimal usability, language inconsistencies, best practice alert lacking patient-specific details, clinicians missing critical information or action, unfamiliarity with the full clinical context, infinite ordering loop leading to error, and having redundant options. The 3 most identified themes were lack of supporting evidence (n=17, 26%), operational barriers arising from the test environment (n=11, 17%), formatting inconsistencies, and lack of following standards (n=8, 12 %; Table 2).

**Table 2.** Thematic analysis distribution.

Theme	Unique issues (n=66)
Lack of supporting evidence	17
Operational barriers arising from the test environment	11
Formatting inconsistencies and lack of following standards	8
Suboptimal data retrieval and display	6
Lack of user control	5
Lacking language clarity	4
Order-set related suboptimal usability	4
Language inconsistencies	3
Best Practice Advisory lacking patient-specific details	2
Clinicians missing critical information, action, or both	2
Unfamiliarity with the full clinical context	2
Infinite ordering loop leading to error	1
Redundant options	1

Lack of supporting evidence refers to the end users’ request to integrate and access evidence-based citations throughout the CDSS. Of the 17 usability issues about evidence-based medicine, 14 were identified by end users and 3 were identified by experts. Examples include users disagreeing with threshold values and requesting evidence-based medicine citations, and users requesting the ability of the CDSS tool to be explained.

Formatting consistency and a lack of following standards refer to the system’s ability to present information clearly and concisely, with a consistent interface to ensure easy readability for the user. Of the 8 usability issues about formatting consistency and standards, 2 were identified by end users, and 6 were identified by experts. Examples include inconsistent and difficult-to-read text usage, style, color, and sizes.

Suboptimal data retrieval and display refer to the system’s ability to present information in a manner that reduces user cognitive burden by increasing smooth and efficient navigation by auto-populating data (such as laboratory results). Of the 6 usability issues about easy retrieval and optimal display, all were identified by end users. Examples include users requesting that laboratory or imaging values and administration time be incorporated into the CDSS.

Lack of user control refers to the flexible design of the system to ensure smooth interaction with the user. Of the 5 usability issues about user control, 2 were identified by end users and 3 were identified by experts. Examples include users requesting the ability to navigate away from the tool and forgiveness when using the CDSS.

Lacking language clarity refers to specifying verbiage to omit any confusion the user may have. Of the 4 usability issues about language clarity, 3 were identified by end users and 1 was identified by experts. Examples include specifying verbiage used throughout the tool and using specific phrases (ie, the use of “active bleed” vs “new bleed” may imply 2 different ideas).

Order set-related suboptimal usability refers to any suboptimal interactions the user may encounter when interacting with the order set. Of the 4 usability issues related to order set-related suboptimal usability, all were identified by end users. Examples include users requesting the tool display prompts regarding its functionality on a case-by-case basis and easy access to the admission order set.

Language inconsistencies refer to the consistent use of verbiage throughout the system. Of the 3 usability issues about language consistency, they were all identified by experts. Examples include discrepancies between verbiage used in the storyboard or title and the consistent use of terms implying the same definition (ie, either “snooze” or “lockout time” should be used, not both).

Best Practice Advisory (BPA) lacking patient-specific details refers to information that the system does not include, which users would prefer to be included. Of the 2 usability issues about BPA lacking patient-specific details, both were identified by end users. Examples include adding patient risk stratification to the BPA (ie, changing the tool to be more diagnostic) and including crucial patient-specific laboratory values.

Clinicians missing critical information refers to ensuring a system design that ensures easy readability for the clinician. Of the 2 usability issues about eliminating the chance of clinicians missing critical information, 1 was identified by end users and 1 was identified by experts. Examples include bolding information and making information more accessible to improve readability.

Unfamiliarity with the context refers to additional guidance presented to the clinician on the system. Of the 2 usability issues about unfamiliarity with context, both were identified by end users. Examples include ensuring proper education and introduction of the tool to clinicians to ensure it does not interrupt their workflow.

A redundant option refers to options already included as part of the system. Of the 1 usability issue about redundant options, it was identified by end users. Examples include omitting the “already ordered complete” button.

An infinite ordering loop leading to error refers to an infinite loop that occurs when using the system, during the beginning of its launch. It has since been resolved as of May 16, 2023. The 1 usability issue about the infinite ordering loop was identified by end users. Examples include users noting their warranted frustration with the ordering process.

Clinicians most often identified issues relating to the readily apparent integration of evidence-based medicine into the CDSS, recognizing a need for the use of citations and principles. Clinicians frequently noted how the CDSS might disrupt their workflow. For practical feasibility, request the integration of critical laboratory or imaging values and consistency in formatting throughout the tool to reduce user cognitive burden and ensure an efficient workflow. A compilation of unique usability issues categorized by theme is included in Multimedia Appendix 1.

### Examples of Usability Modifications

**Figure 5.**
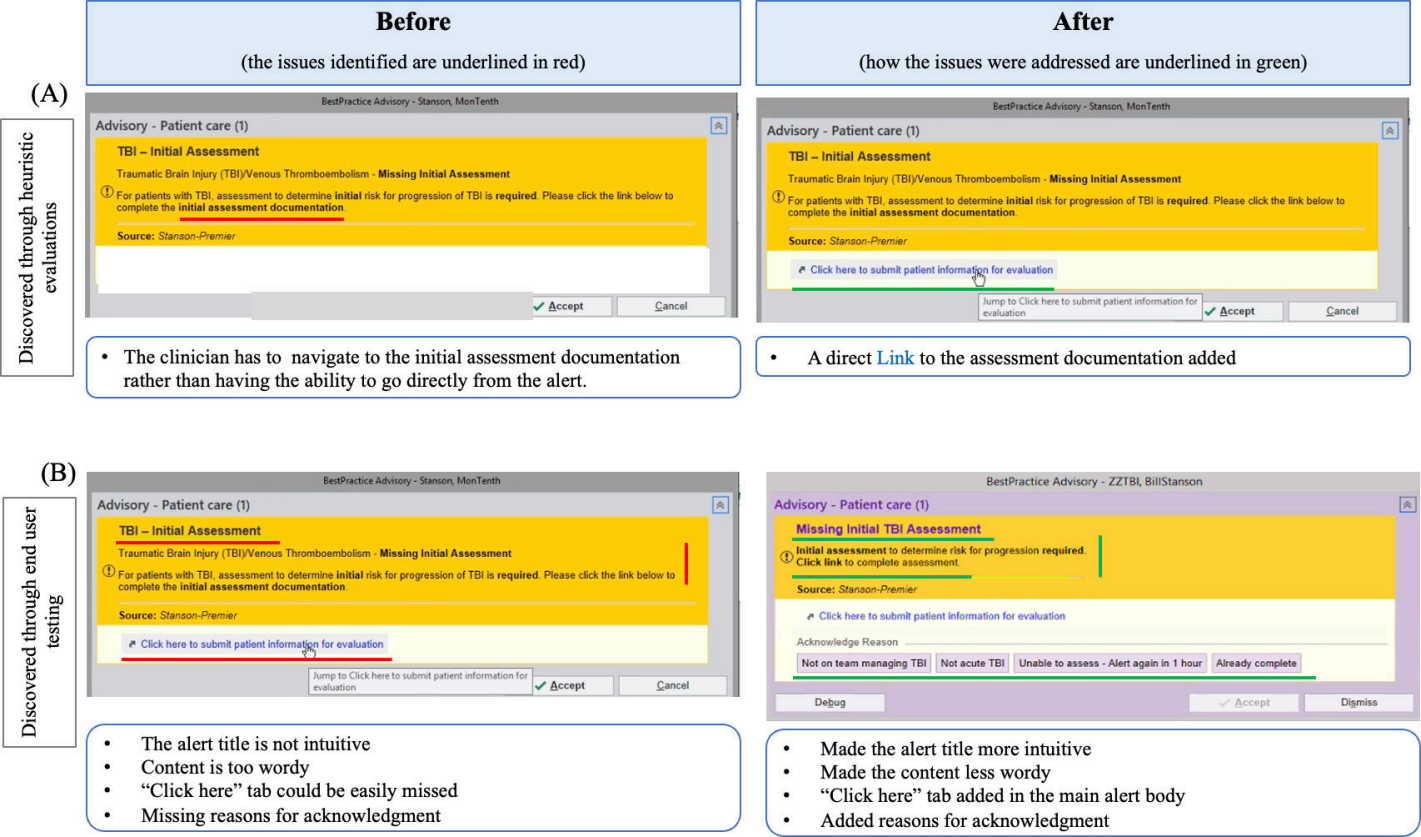
Examples of modifications. TBI: traumatic brain injury. Copyright 2024 Epic Systems Corporation. Used with permission.

Figures5  6 present a few examples of modifications made based on insights from heuristic evaluations and usability testing by end users. The full list of identified usability issues is provided as a supplementary table.

**Figure 6.**
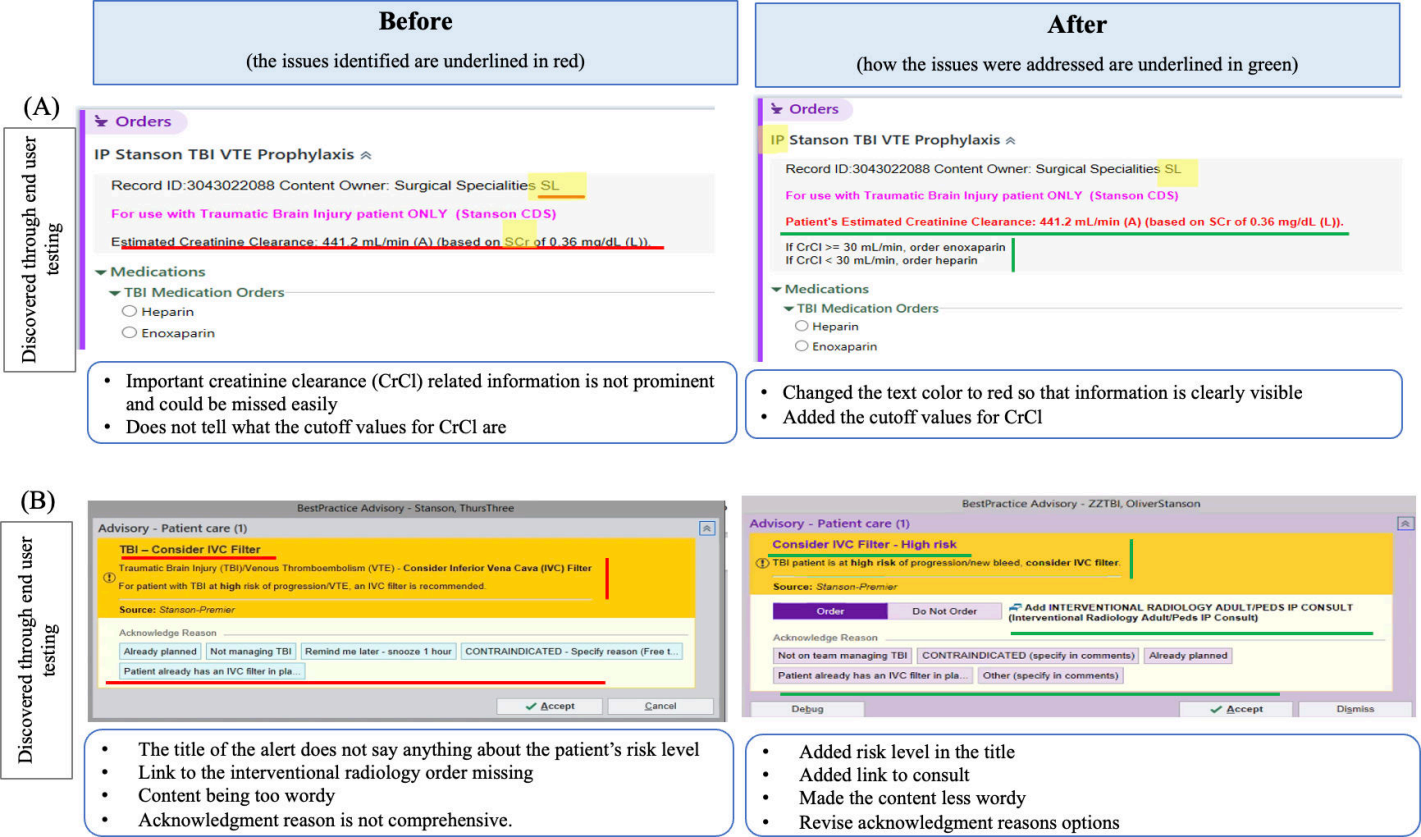
Examples of modifications. Images included with approval from Epic. CrCl: creatinine clearance; IP: inpatient; IVC: inferior vena cava; SL: service line; SCr: serum creatinine; T/PEDS: ADULT/PEDS; TBI: traumatic brain injury; VTE: venous thromboembolism. Copyright 2024 Epic Systems Corporation. Copyright 2024 Epic Systems Corporation. Used with permission.

## Discussion

### Summary and Interpretation of Findings

Using a dual usability evaluation approach, namely expert-driven heuristic evaluation and end user–driven usability testing, we identified a variety of both similar and unique issues across the traumatic brain injury venous thromboembolism CDSS. Of the 66 unique issues identified, the percentage attributed to each method was as follows: usability testing (43 issues, 65%), heuristic evaluations (17 issues, 26%), and both methods together (6 issues, 9%). Although the mean severity scores of the identified problems did not differ significantly between the 2 methods, issues that were ranked as severe and catastrophic were exclusively found through usability testing by the end users. The most common issues identified across both methods were related to a lack of supporting evidence, operational barriers, and formatting consistency or lack of following standards. Heuristic evaluations primarily identified issues related to formatting consistency or lack of following standards, user control, suboptimal language clarity, and consistency. In contrast, usability testing revealed a broader range of issues, where lack of supporting evidence, operational barriers, and suboptimal data retrieval and display were found to be the most common.

Using a combination of these 2 methods can identify a wider range of usability issues. This aligns with prior evidence suggesting that combining 2 methods of usability evaluation can facilitate the development of an optimal CDSS [24]. We found that the majority of issues identified by usability testing were only discovered by end users, as compared to the experts. Although the experts identified fewer issues, they pinpointed more granular issues related to the layout, formatting, and consistency. In contrast, end users were more concerned with larger, more consequential issues, such as the lack of supporting evidence to increase their trust and confidence and fragmented workflow (primarily sending form test environment-related constraints). The findings from this study validate many of the claims made in previous comparisons of heuristic evaluations and usability testing [31,32] such as heuristic evaluations being more cost-effective in terms of time and resources, and the ability of each method to identify distinct sets of usability issues with very small convergence rates (9% in our study). Heuristic evaluation findings were limited to identifying issues, whereas end users engaged in usability testing provided both strengths and weaknesses. For example, end users appreciated the clarity of the premier questionnaire (which covered the critical issue for risk profiling), as well as the straightforward alert verbiage. They also appreciated that the creatinine clearance value was apparent in the laboratory order results, eliminating the need to navigate to find it.

### Limitations

There are some limitations to this study. First, the usability evaluation was conducted at a single institution (M Health Fairview) using a single electronic health system (Epic). We had 5 clinicians participate in the end user testing. Several usability evaluation resources suggest that testing with 5 individuals is often considered appropriate for identifying usability issues, especially in qualitative analysis [26]. However, a larger sample size would have strengthened the generalizability of the findings for quantitative analysis. Additionally, we did not examine whether participants’ amount of experience in their respective fields or their overall familiarity with digital technology influenced their insights.

Usability testing being conducted in the Epic test environment introduced some constraints that users had to adjust to, such as manually shortened refresh time frames to decrease testing time, which resulted in some unusual system behaviors. To simulate the passage of time, users had to right-click anywhere and hit “refresh” after responding to alerts. For the testing session, test results and imaging reports were simplified and appeared shorter in length. Consequently, the insights gathered during the simulation may not completely represent the realities and challenges faced by clinicians working in high-pressure environments, caring for their patients who are critically ill. Finally, the IT team faced challenges due to system constraints, as the systems were hardwired with limited ability for upgrades. We were, however, able to incorporate several build-related upgrades through close collaborations. We are still in the process of upgrading the tool based on the observations made during our evaluations and the feedback collected from end users since the tool went live.

### Implications for Future Directions

Concerning further CDSS implementation in different hospital settings, we plan to scale and assess the effectiveness of the CDSS for venous thromboembolism prophylaxis guidelines in patients with traumatic brain injury. This will consist of a stepped wedge hybrid effectiveness implementation trial to scale the CDSS across 9 sites within 4 trauma systems.

### Conclusions

By using dual usability evaluation methods, leveraging both expert and end user input during the early phases of system development in an iterative manner, we were able to identify a diverse range of usability issues. These issues varied in severity and were relevant to each evaluator based on their respective experience and role. Through conducting this study and our continued efforts to gather feedback from end users, we have been able to optimize the design and functionality of the traumatic brain injury–CDSS to align more effectively with clinicians’ needs and workflow. This comprehensive approach has provided us with valuable insights in refining CDSS, ultimately improving its usability and effectiveness in clinical practice.

## Supplementary material

10.2196/60268 Multimedia Appendix 1 Usability issues by theme.

## Abbreviations

BPA: Best Practice Advisory
CDSS: clinical decision support system
CPG-FHIR: clinical practice guidelines on Fast Healthcare Interoperability Resources
*ICD-10-CM*: *International Statistical Classification of Diseases, Tenth Revision, Clinical Modification*
IT: information technology
SEQ: Single Ease Question
SUS: Systems Usability Scale
UMN: University of Minnesota
