# Supplementary material for: Leveraging Dual Usability Methods to Evaluate Clinical Decision Support Among Patients With Traumatic Brain Injury: Mixed Methods Study
Source: JMIR Hum Factors. 2025 Jul 30;12:e60268. doi: 10.2196/60268 (PMC12310145; doi:10.2196/60268)
Supplement: Multimedia Appendix 1 — Usability issues by theme. [file humanfactors-v12-e60268-s001.docx]

**Table S1.** Usability issues by theme.

| **Theme** | **Usability Issue** | **CDSS Element** | **Method of Identification** | **Severity Score** |
| --- | --- | --- | --- | --- |
|  |  |  |  |  |
| Evidence-based medicine (EBM) |  |  |  |  |
|  | User requests prognostic tool built in, i.e., a tool that anticipates potential outcomes for the patient. Requests title of tool prompts renamed for clarity | Initial/ daily assessment | *E-UT | 1 |
|  | User requests feedback after completing questionnaire. Requests a visual (logic tree) surrounding the assessment of the algorithm | Daily assessment | E-UT | 2 |
|  | User unsure about the value of the tool, i.e., how beneficial it is for the care team and their patients. User wants to ensure that time spent interacting with the CDSS will have beneficial patient outcomes | General | E-UT | 3 |
|  | Patient progress, i.e., when the alert was fired, is not displayed in the TBI VTE prophylaxis pathway | Daily assessment | **HE | 2 |
|  | No documentation on the design and functionality of the CDSS present | Initial/ daily assessment | HE | 2 |
|  | Disagreement with utilization of creatinine clearance. User requests creatinine | Lab order alert | E-UT | 1 |
|  | User requests addition of GFR to be utilized with creatinine clearance | Lab order alert | E-UT | 1 |
|  | User requests EBM citations for IVC filter, stating the implementation of an IVC filter requires team heavy discussion and recommendation | Daily assessment | E-UT | 4 |
|  | User disagrees with 30 BID enoxaparin, stating they use 40 mg daily. User disagrees with ordering Anti-Xa level | Anticoagulation alert | E-UT | 2 |
|  | User requests EBM citations for ordering enoxaparin, citing skepticism | Anticoagulation alert | E-UT | 2 |
|  | Repeat head CT alert not issued for patient after 48 hours. User requests EBM citations for 48-hour guideline | Follow up CT scan alert | E-UT | 2 |
|  | Link to EBM citations for Anti-Xa indications should be included | Lab order alert | HE | 2 |
|  | User states they normally don’t check Anti-Xa levels for Prophylaxis dosing | Lab order alert | E-UT | 2 |
|  | User notes discrepancy in checking Anti-Xa level for enoxaparin but not for Heparin. Requests EBM citations | Lab level alert/ anticoagulation alert | E-UT | 2 |
|  | User expresses discontent with Anti-Xa range being equivalent of low intensity Heparin drip | Lab level alert/ anticoagulation alert | E-UT | 1 |
|  | User requests more clarity on what the CDSS is recommending | Initial/ daily assessment | E-UT | 3 |
|  | User requests explicit instructions added to CDSS. Increasing Enoxaprin by 10 mg should be changed to increasing by 40 mg | Anticoagulation alert | E-UT | 2 |
| Formatting consistency  & standards |  |  |  |  |
|  | TBI acronym is spelled out in the second line of the alert, but not spelled out in the alert title | General alert | HE | 2 |
|  | Too much text in BPA. User requests succinct language | General alert | E-UT | 1 |
|  | Title of missing VTE Prophylaxis and monitoring is cut off on storyboard alert | General alert | HE | 2 |
|  | Word count limit in textbox (other) missing | General alert | HE | 1 |
|  | Size of font used in BPA is small | General alert | HE | 1 |
|  | The word initial is capitalized (INITIAL). The words following in the phrase are in lower case (assessment, documentation) | Initial assessment | HE | 1 |
|  | Use of font color to make CrCl value stand out | Lab order alert | E-UT | 2 |
|  | Change the selection of standard alert colors based on urgency of required action | General alert | HE | 2 |
| Easy retrieval & optimal  display |  |  |  |  |
|  | User asks if it is necessary to enter the birth date every time with each new assessment completed | Daily assessment | E-UT | 3 |
|  | User notes verifying Xa 4 hours after the 4^th^ dose is cumbersome. Requests an efficient way to view the results | Lab level alert | E-UT | 3 |
|  | User unable to find the exact time patient arrived at the ED | Initial assessment | E-UT | 3 |
|  | User requests the CDSS incorporate current orders placed when firing an alert. User suggests if they order a scan not necessary, the tool should fire an alert stating “Are you sure you want to order this? This was done X time ago” with result time/date auto populating in the alert | Follow up CT scan alert | E-UT | 3 |
|  | User prefers to manually check CrCl through lab tab, instead of chart review, which shows the value is unable to be calculated. Notes the benefit of manually checking is viewing the GFR before placing orders | Lab order alert | E-UT | 2 |
|  | User requests the CDSS includes the time a dose was last administered and the timing of the next dose | Lab level alert | E-UT | 3 |
| User control |  |  |  |  |
|  | User states STAT CT order is problematic, should only be used in emergent situations | Follow up CT scan alert | E-UT | 2 |
|  | No undo button to redo selection when filling out assessment | Initial/ daily assessment | HE | 2 |
|  | User must navigate away from BPA to discontinue orders | Discontinue anticoagulation order | E-UT | 2 |
|  | Initial assessment documentation should be linked directly to assessment documentation | Initial assessment | HE | 2 |
|  | No option to select more than one option under “acknowledge reasons” | Initial/ daily assessment | HE | 2 |
| Language clarity |  |  |  |  |
|  | Active bleed vs new bleed phrase in the questionnaire should be more specific to ensure that nothing new has evolved since patient last seen | Initial/ daily assessment | E-UT | 2 |
|  | Specify verbiage of last CT completed in relation to when the CDSS alert fired | Follow up CT scan alert | E-UT | 1 |
|  | Specify definition of missing initial assessment to avoid confusion | Initial assessment | E-UT | 2 |
|  | Specify if “acknowledge reasons” is a command | General alert | HE | 1 |
| Order set related |  |  |  |  |
|  | Reason for IVC consult in order set is not visible unless location (cascading) is clicked. Location should be a hard stop | Discontinue anticoagulation order | E-UT | 2 |
| Suboptimal usability |  |  |  |  |
|  | User requests the CDSS has prompts built into the system to remind the user of the tool’s functionality, including when anticoagulation is appropriate to start on a case-by-case basis | Initial/ daily assessment | E-UT | 3 |
|  | Admission order set requires entering attending’s name three times | Initial assessment | E-UT | 3 |
|  | User unable to determine if patient is intubated | Initial assessment | E-UT | 2 |
| Language  consistency |  |  |  |  |
|  | Lockout time when already documented should say “forever” | General alert | HE | 2 |
|  | Use of “snooze” vs “lockout time” | General alert | HE | 2 |
|  | Discrepancies in verbiage for storyboard and title present | General alert | HE | 1 |
| BPA lacking patient.  specific details |  |  |  |  |
|  | Patient risk stratification should be clear in BPA, i.e., patient at low risk of progression based on questionnaire results | Daily assessment | E-UT | 2 |
|  | BPA does not have lab levels included | Lab level alert/ anticoagulation alert | E-UT | 2 |
| Eliminating chance of clinicians missing  critical information |  |  |  |  |
|  | User states when admitting a patient, their instinct is to click accept on the multiple window pop ups they receive. User suggests moving the blue text on top of the accept button, and to change the wording to “required step” to alert clinicians to not ignore the tool | Initial/ daily assessment | E-UT | 2 |
|  | Clinician could miss important things due to poor readability. Mitigate this by bolding critical information | General alert | HE | 2 |
| Unfamiliarity with context |  |  |  |  |
|  | User notes storyboard alert is often ignored by residents, as it is not part of their routine workflow | Daily assessment | E-UT | 2 |
|  | User concerned about what other services have access to the CDSS. Requests the tool is restricted based on login department | General alert | E-UT | 2 |
| Redundant option |  |  |  |  |
|  | User states “already ordered complete” button should not be listed | Lab order alert | E-UT | 3 |
| Infinite ordering loop leading to error |  |  |  |  |
|  | User states feedback loop is frustrating, which will be problematic until fixed by Epic. Notes the frustration will lead people to not utilize the tool | Follow up CT scan alert | E-UT | 4 |

**E-UT: End user testing; **HE: Heuristic evaluations: A 0 to 4 rating scale was used to rate the severity of usability problems (0 = I don't agree that this is a usability problem at all; 1 = Cosmetic problem only: need not be fixed unless extra time is available on the project; 2 = Minor usability problem: fixing this should be given low priority; 3 = Major usability problem: important to fix, so should be given high priority; 4 = Usability catastrophe: imperative to fix this before the product can be released)*
